# SIRT3 activation promotes enteric neurons survival and differentiation

**DOI:** 10.1038/s41598-022-26634-9

**Published:** 2022-12-21

**Authors:** Arun Balasubramaniam, Ge Li, Anita Ramanathan, Simon Musyoka Mwangi, C. Michael Hart, Jack L. Arbiser, Shanthi Srinivasan

**Affiliations:** 1 Division of Digestive Diseases, Emory University School of Medicine, Atlanta, GA USA; 2 Department of Dermatology, Emory University School of Medicine, Atlanta, GA USA; 3 Division of Pulmonary, Allergy, Critical Care and Sleep Medicine, Emory University School of Medicine, Atlanta, GA USA; 4 Atlanta Veterans Affairs Health Care System, Decatur, GA USA

**Keywords:** Cell death in the nervous system, Diseases of the nervous system

## Abstract

Enteric neuron degeneration has been observed during aging, and in individuals with metabolic dysfunction including obesity and diabetes. Honokiol, a naturally occurring compound, is an activator of Sirtuin-3 (SIRT3) that has antioxidant activity. Its role in modulating enteric neuron-specific neurodegeneration is unknown. We studied the effects of honokiol and its fluorinated analog, hexafluoro-honokiol, on enteric neuronal differentiation and survival. We used a previously established model of mouse primary enteric neuronal cells and an enteric neuronal cell line treated with palmitate (PA) and lipopolysaccharide (LPS) to induce mitochondrial dysfunction and enteric neuronal cell death. The effect of honokiol and hexafluoro-honokiol was assessed on neuronal phenotype, fiber density, differentiation, and pyroptosis. Honokiol and hexafluoro-honokiol significantly increased neuronal networks and fiber density in enteric neurons and increased levels of neuronal nitric oxide synthase and Choline acetyltransferase mRNA. Hexafluoro-honokiol and honokiol also significantly increased SIRT3 mRNA levels and suppressed palmitate and LPS-induced neuronal pyroptosis. SIRT3 knock-down prevented the hexafluoro-honokiol mediated suppression of mitochondrial superoxide release. Our data supports a neuroprotective effect of honokiol and its derivative and these could be used as prophylactic or therapeutic agents for treating enteric neurodegeneration and associated motility disorders.

## Introduction

The enteric nervous system (ENS), also known as the “second brain”, is a complex and integrative center that is present in the gastrointestinal tract^1,2^. The ENS comprises complex neuronal networks, cells, and transmitters that modulate critical gastrointestinal functions including digestion, nutrient absorption, motility, and energy balance, independently from the brain and the spinal cord. However, the ENS and the central nervous system (CNS) coordinate and communicate with each other using the sympathetic and parasympathetic nervous systems, endocrine systems, immune cells, and the gut microbiome to maintain gut homeostasis^1–3^. The ENS comprises neurons and glial cells within the neuronal ganglia in two major nerve centers—the myenteric plexus that controls smooth muscle cells (SMCs), interstitial cells of Cajal (ICC), and platelet-derived growth factor α PDGFRα + cells, which together make up the systemic infection and proliferation (SIP) syncytium, integrate their behavior to produce smooth muscle contractions, and relaxation in gut motility. The submucosal plexus modulates absorption, secretion, and blood flow in the gut^4,5^. These two plexuses work in harmony to control normal gastrointestinal tract function.

ENS degeneration can lead to many disorders related to altered motility and inflammation. These include primary achalasia^6,7^ and irritable bowel syndrome (IBS)^8,9^. Changes in the ENS have also been noted in association with CNS disorders ranging from neurodevelopmental (autism disorders) to neurodegenerative (Parkinson’s and Alzheimer’s diseases)^10^, and metabolic disorders such as obesity and diabetes^10,11^. Studies^12^ suggest that diseases like Parkinson’s disease might begin and show pathological symptoms in the ENS before it manifests in the CNS. Oxidative stress in the ENS stimulated by endogenous and exogenous factors, although advantageous at steady-state levels, becomes toxic to neurons at higher levels. The persistent generation of reactive oxygen species (ROS) can cause oxidative stress and injure nitrergic and cholinergic enteric neurons, especially during aging, disease, and inflammation. Mitochondria, a common source of ROS generation, are implicated in neurodegeneration^13^. Thus, developing and investigating therapeutics that target oxidative mitochondrial dysfunction offers a novel strategy for ENS protection.

Honokiol, a naturally occurring polyphenol extracted from the bark of the magnolia tree *Magnolia grandiflora* that is endemic to Asia, is used in traditional Asian medicines^14^. Previous studies have demonstrated that honokiol, an antitumor and antiangiogenic agent^15^, and hexafluoro-honokiol, a novel fluorinated synthetic honokiol analog, exert a protective effect against melanoma in vivo^16^ and enhance SIRT3 expression^17^. Honokiol also demonstrated an anti-inflammatory effect in mice with diarrhea induced by enterotoxin by promoting the intestinal barrier and regulating apoptosis of the intestinal epithelium^18^. An advantage of honokiol is that it exerts a direct effect on neuronal cells in the CNS by crossing the blood–brain barrier easily^19^. A combinatorial administration of honokiol alone or with other compounds showed a neuromodulating and neuroprotective effect on the rodent model for diseases related to the CNS and peripheral nervous system^20^. The pleiotropic effects of honokiol in the CNS^14,16,19,21^ and gastric mucosa^18,20^ suggest a potential role for honokiol in the ENS.

The mechanisms for the therapeutic effects of honokiol continue to be defined. Previous models showed that honokiol activates key proteins like SIRT3 and its downstream targets such as peroxisome proliferator-activated receptor γ coactivator 1α (PGC-1α) to counter the production of ROS that are induced under stress/injury^22^. The reduced levels of SIRT3 owing to aging, diseases, or poor lifestyle habits^23^ as well as knock out of SIRT3 in experimental models of various diseases^23,24^ show increased oxidative damage and severe alterations in the acetylation status of mitochondrial proteins, emphasizing the role of SIRT3 in maintaining mitochondrial homeostasis under stress^25^. A key target of SIRT3 is superoxide dismutase-2 (SOD2). In response to oxidative stress, SIRT3-dependent deacetylation of SOD2 is a novel post-translational regulation at lysine 68 (K68) and 122 (K122) which enhances enzymatic activity^26–29^. Oxidative stress-induced SOD2 expression is believed to be a chief cellular defense mechanism^30^. As SIRT3 activity decreases, SOD2 gets inactivated by acetylation leading to enhanced oxidative stress. In the case of the ENS, the role of SIRT3 in protecting neurons against oxidative stress has not been investigated. In this study, we investigated if honokiol can enhance the survival and differentiation of primary myenteric neurons and in vitro cultured neuronal cells and if the neuroprotective activity of honokiol is mediated by SIRT3.

## Materials and methods

### Animals

This study followed ARRIVE guidelines on the use of experimental animals. All animal experiments were approved by the Atlanta Veteran Affairs Medical Center Institutional Animal Care and Use Committee and conducted according to recommended guidelines.

### Primary enteric neuronal cell isolation and culture

Myenteric neurons were isolated as previously described^31^ from whole intestines from 8–12 weeks old male C57BL/6 J mice. The cells were resuspended in Neurobasal A Medium (#10,888,022, Gibco, Grand Island, NY, USA) supplemented with 2 mM L-glutamine (#25,030,081, Gibco), B27 (#A3582801, Gibco), penicillin/streptomycin (#15,140,122, Gibco), and 10 ng/mL Glial cell line-derived neurotrophic factor (GDNF, #200–37, Shenandoah Biotechnology, Warwick, PA, USA) and 1% FBS (#S10650H, Atlanta Biologicals, Atlanta GA, USA) and seeded, respectively, at a density of 1 × 10^4^ and 1 × 10^5^ cells per well into Matrigel-coated 4 well tissue culture chamber slides and 6 well tissue culture plates. The cells were placed at 37 °C in a humidified incubator with 5% CO_2_ as described previously^31^, and the growth medium was replaced after 24 h. Once the cells are confluent, a medium containing either honokiol (10 µM)^32^ or hexafluoro-honokiol (10 µM)^32^ or 0.1% ethanol (vehicle) was used as a treatment. These concentrations were used based on previously published studies.

### Culture and treatment of mouse enteric neuronal cell line

Immortal mouse fetal enteric neuronal (IM-FEN) cell line^33^ cells were plated in a modified N2 medium containing GDNF (10 ng/ml) (Shenandoah Biotechnology), 10% fetal bovine serum (Atlanta Biologicals), and 20 U/ml of recombinant mouse interferon-γ (Chemicon, #IF005 ) and cultured at 33 °C in a humidified tissue culture incubator with 5% CO_2_. After 24 h, the medium was changed to Neurobasal-A medium (Gibco) containing B-27 Plus Supplement (Gibco), 1 mmol/L glutamine (Gibco), 1% fetal bovine serum, GDNF (10 ng/ml), and the cells were transferred to a 39 °C incubator containing 5% CO_2_. After 5 days, the cells were treated with or without PA (0.5 mM) (Sigma-Aldrich, St. Louis, MO) LPS (0.5 ng/ml) (Sigma), and hexafluoro-honokiol (10 µM) and cultured for a further 24 h.

### Immunofluorescence staining and imaging of myenteric neurons

Neuronal cells were fixed for 20 min at room temperature in 4% paraformaldehyde in phosphate-buffered saline (PBS) and permeabilized at 4 °C for 15 min with 0.3% Triton-X 100. The cells were then blocked with 5% Bovine serum albumin (BSA) in PBS for 1 h and incubated at 4 °C with gentle shaking with a 1:1000 dilution of mouse anti-β-III-tubulin (Tuj-1) antibody (#ab78078, Abcam, Waltham, MA, USA) or 1:400 dilution of rabbit anti-nNOS antibody (#ab76067, Abcam) in blocking buffer. After overnight incubation, the cells were incubated for 1 h at room temperature with a 1:200 dilution of Alexa Fluor 488 conjugated donkey anti-rabbit IgG secondary antibody (#A-21206, Molecular Probes, Eugene, OR, USA) or 1:400 dilution of Alexa Fluor 594 conjugated anti-rabbit IgG secondary antibody (#A-21207, Molecular Probes) before the nuclei were labeled with 4,6-diamidino-2-phenylindole (DAPI, #D3571, Molecular Probes). They were then mounted in Prolong Gold antifade mounting medium (#P36930, Invitrogen, Eugene, OR, USA) and visualized with the aid of an Olympus IX51 microscope (Olympus, Tokyo, Japan) equipped with the cellSens Standard 1.12 imaging software (Olympus) for fluorescence imaging or Nikon A1R (Nikon Instruments, Melville NY, USA) issued with NIS Elements software for confocal imaging. Neuronal cells were scored for fiber length, the number of nNOS-positive cells, and number of DAPI cells using ImageJ software^34^. At least 8–10 fields were examined per group and all experiments have been replicated a minimum of three independent times.

### Quantitative real-time polymerase chain reaction (qRT-PCR)

Total RNA was isolated using the RNeasy Mini kit (Qiagen, Hilden, Germany) and first-strand cDNA was synthesized using the SuperScript VILO Mastermix kit (Invitrogen, Carlsbad, CA, USA). Real-time PCR reactions were set up using Fast SYBR Green Master Mix (Applied Biosystems, Foster City, CA, USA) and oligonucleotide primers specific for the mouse genes (Table 1). Thermal cycling was performed on a StepOnePlus Real-Time PCR System (Applied Biosystems). The levels of nNOS, ChAT, and SIRT3 mRNAs were normalized to the mRNA levels of the housekeeping gene GAPDH to allow comparisons among the different experimental groups using the delta Ct method^35^.

**Table 1:** List of oligonucleotide primers used in this study.

Mouse gene	Oligonucleotide primer
nNOS	Upstream, 5′-GCCTTCAAGTACTACCTGGACA-3′; Downstream 5′-CTCATATTCCTGAAGCCCCTTG-3′
ChAT	Upstream, 5′- ATGCAACACCTGGTACCTGAAG-3′; Downstream, 5′-CAGCCAGTATTCAGAGACCCAA-3′
SIRT3	Upstream, 5′- GGCCCTGCCCTTGAGGCATTAAA-3′; Downstream, 5′- CCACCTGTAACACTAGTCCTCGCCA-3′
GAPDH	Upstream, 5′-TTGTGATGGGTGTGAACCACGA-3′; Downstream, 5′-TCTTCTGGGTGGCAGTGATGG-3′

### Western blotting

Neuronal cells were lysed in 1 × Laemmli sample loading buffer (Bio-Rad, Hercules, CA, USA) supplemented with complete protease inhibitor cocktail (Roche Diagnostics, Mannheim, Germany) and the proteins separated on 4–20% Criterion TGX gradient gels (Bio-Rad) according to recommended procedure. Separated proteins were transferred onto Immune-Blot polyvinylidene difluoride (PVDF) membranes (Bio-Rad) according to recommended procedure. The membranes were then probed with primary rabbit antibodies to nNOS (#ab78078, Abcam), cleaved caspase-1 (#4199, Cell Signaling Technology, Boston, MA), SIRT3 (#5490, Cell Signaling Technology) diluted 1:1000, and mouse monoclonal antibodies to α-tubulin (#3873S, Cell Signaling Technology) and β-actin (#a5441, Sigma-Aldrich, St. Louis, USA) diluted, respectively, 1:1000 and 1:5000. Horseradish peroxidase-conjugated anti-mouse and anti-rabbit IgG (Cell Signaling Technology) secondary antibodies were used at a 1:2000 dilution. All semi-quantitative measurement of band intensity was performed using the ImageJ analysis software (US National Institutes of Health, Bethesda, Maryland, USA).

### siRNA transfection

Neuronal cells grown for at least 24 h in a complete cell culture medium without antibiotics were seeded at recommended densities in tissue culture plates and reverse transfected with 50 nM ON-TARGETplus SMARTpool Mouse SIRT3 siRNA (Horizon Discovery, Cambridge, United Kingdom) or ON-TARGETplus Non-targeting Control siRNA (Horizon Discovery) using Lipofectamine RNAiMAX Reagent (Invitrogen, Carlsbad, CA, USA). The transfection media were replaced after 24 h with a complete medium supplemented with antibiotics and with or without honokiol, hexafluoro-honokiol, BSA-conjugated palmitate, and LPS. The transfected cells for total RNA were isolated for gene expression analyses and MitoSOX assay for superoxide measurement was used after 24 h while proteins were harvested for Western blotting after 48 h.

### Assay for mitochondrial ROS

Neuronal cells were cultured in 96 well plates at 80% confluency with and without SIRT3-siRNA transfection, then incubated for 10 min in the dark in a 37 °C incubator loaded with 5 µM MitoSOX (#M36008, Invitrogen) reagent working solution. The cells were washed and counterstained with Hoechst 33,342 (#62,249, Thermo Scientific, Rockfold, IL, USA) in fixation solution 15 min at room temperature. After two washes with PBS the cells imaged on a Olympus IX51 microscope (Olympus).

### Statistical analysis

GraphPad Prism 9.0 (https://www.graphpad.com/; GraphPad Software, La Jolla, CA, USA) software was employed for data analysis by unpaired t-test and 2-way ANOVA as appropriate. The significant difference was considered by P-values observation as follows: *P*-values of < 0.05 (*), < 0.01 (**), and < 0.001 (***). The mean ± standard error of the mean (SEM) was obtained from at least three separate experiments.

## Results

### Honokiol and hexafluoro-honokiol increase fiber density, nNOS, and ChAT neurons in enteric neuronal cells

**Figure 1:**
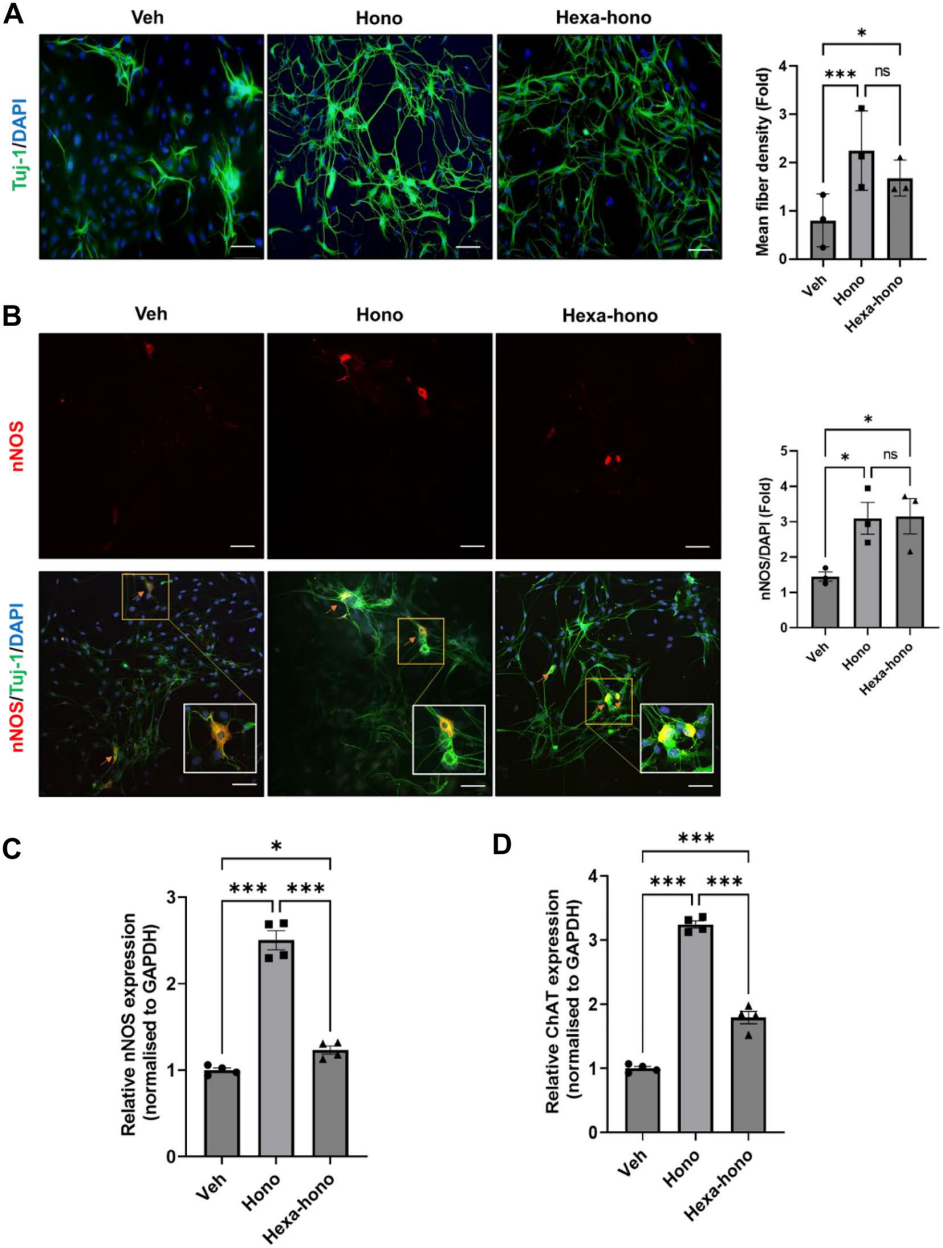
Honokiol and hexafluoro increase fiber density, nNOS, and ChAT neurons in primary myenteric neuronal cells (**A**) Representative fluorescent microscopy images of primary myenteric neurons treated with vehicle, Honokiol (10 μM), or Hexafluoro-honokiol (10 μM), for 24 h, and stained with neuronal marker, Tuj-1, and the nuclear stain 4,6-diamidino-2-phenylindole (DAPI). Scale bar = 50 μm. The bar graph shows the neuronal fiber density with respect to the total number of cells in each field (**B**) Representative confocal microscopy images of primary myenteric neurons stained for nNOS. Scale bar = 50 μm. The histogram represents the percentage of the number of nNOS neurons with respect to the total number of DAPI-stained cells. (**C**) Honokiol and Hexafluoro-honokiol significantly increased mRNA expression for nNOS and ChAT in primary myenteric neurons. Quantitative RT-PCR was done using primary myenteric neurons treated with vehicle, Honokiol, or Hexafluoro-honokiol for 24 h. The histograms show a fold increase with respect to the vehicle of nNOS or (**D**) ChAT expression. Data are the mean ± SEM from three independent experiments. ns = non-significant; *P < 0.05; ***P < 0.001; (2-way ANOVA).

Previous reports have established the role of honokiol as a potent agent against microbes, inflammation, tumors, and oxidative stress, in different organs but their effect on the ENS has not been thoroughly investigated^36,37^. We examined the effect of honokiol and its fluorinated analog, hexafluoro-honokiol on the differentiation of primary enteric neurons by visualizing the neuronal network using immunofluorescence and by calculating fiber density. Neuronal differentiation was assessed by neural fiber branching and outgrowth represented as neuronal density^38,39^. Cells were cultured for 5 days and treated with vehicle, honokiol (10 μM), or hexafluoro-honokiol (10 μM). At the end of the experiment, cells were stained with β-III-tubulin (Tuj-1), a neuronal marker, and 4,6-diamidino-2-phenylindole (DAPI) to stain the nuclear region. Honokiol and hexafluoro-honokiol promoted enteric neuronal differentiation significantly as seen by increased fiber density (honokiol, 2.26 ± 0.37 fold, *P* < 0.001; hexafluoro-honokiol, 1.62 ± 0.14 fold, *P* < 0.01) compared with vehicle-treated neurons (Fig. 1A). We next examined the effect of honokiol and hexafluoro-honokiol on the expression of neuronal subtypes in primary enteric neurons using confocal microscopy and quantitative RT-PCR. Nitrergic neurons release nitric oxide (NO) as a neurotransmitter, which has an inhibitory function and relaxes gastrointestinal smooth muscle. Electric field stimulation (EFS) causes NO to be released from the myenteric plexus^40^. Honokiol and hexafluoro-honokiol induced the expression of both the inhibitory and excitatory neurotransmitters: neuronal nitric oxide synthase (nNOS) neuronal numbers (honokiol, 3.184 ± 0.428 fold, *P* < 0.05; hexafluoro-honokiol, 3.15 ± 0.501 fold, *P* < 0.01) and mRNA expression (honokiol, 2.50 ± 0.11 fold, *P* < 0.001; hexafluoro-honokiol, 1.23 ± 0.04 fold, *P* < 0.05) normalized to a vehicle (Fig. 1B, C) and increased choline acetyltransferase (ChAT) mRNA expression (honokiol, 3.24 ± 0.055 fold *P* < 0.001, hexafluoro-honokiol, 1.79 ± 0.09 fold, *P* < 0.05) relative to vehicle (Fig. 1D). These results suggest that both honokiol and hexafluoro-honokiol can augment enteric neuronal cell differentiation and stimulate neurotransmitter expression.

### Hexafluoro-honokiol enhances nNOS neurons and protein expression under stress

**Figure 2:**
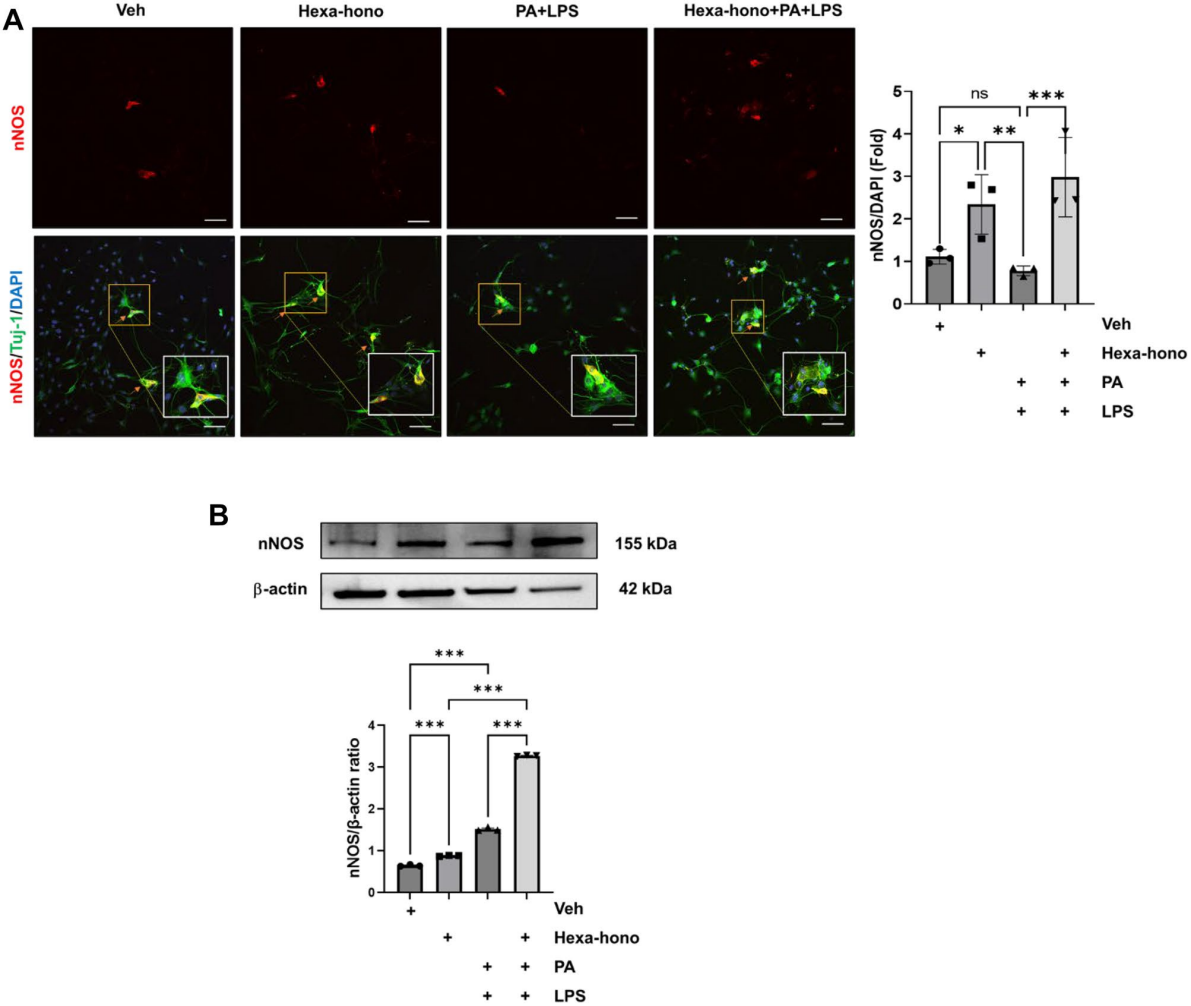
Hexafluoro-honokiol increased nNOS neurons and expression in primary myenteric neuronal and IM-FEN cells treated with Palmitate and LPS. (**A**) Representative confocal microscopy images of primary myenteric neurons treated with Vehicle, Hexafluoro-honokiol (10 μM), PA (0.5 mM) with LPS (0.5 ng/ml), and Hexafluoro-honokiol with PA and LPS, cultured for 24 h, and probed for nNOS, Tuj-1, and DAPI as a nuclear stain. The histogram represents the percentage of the number of nNOS neurons with respect to the total number of DAPI-stained cells. (**B**) Western blot was run with protein lysates from IM-FEN cell lysate probed for nNOS and β-actin as a loading control. The histogram shows nNOS band density normalized to β-actin. The uncropped Western blot images of Fig. 2B has been provided in the supplementary file. Both protein of interest and loading control were run on a single membrane. Data are the mean ± SEM from three independent experiments. ns = non-significant; *P < 0.05; **P < 0.01; ***P < 0.001; (2-way ANOVA).

We have previously demonstrated that PA and LPS can potentially damage enteric neurons in vitro^41^. We investigated whether hexafluoro-honokiol, an equipotent and easier to extract compound than honokiol and a key enhancer of SIRT3^17^ could prevent the neuronal damage induced by PA and along with lipopolysaccharide (LPS) on the primary myenteric neuronal and IM-FEN cells. Primary myenteric neuronal cells were cultured for 5 days and treated with vehicle, hexafluoro-honokiol (10 μM), PA (0.5 mM) with LPS (0.5 ng/ml), and Hexafluoro-honokiol with PA and LPS treated for 24 h, and probed for nNOS, Tuj-1, and DAPI markers. nNOS neurons significantly increased with the treatment of hexafluoro-honokiol. PA and LPS reduced nNOS neurons, although this was not statistically significant with the vehicle, this effect was reversed by hexafluoro-honokiol treatment (hexafluoro-honokiol, 2.34 ± 0.370 fold, *P* < 0.05; PA + LPS, 0.78 ± 0.21 fold, ns; Hexa + PA + LPS, 2.98 ± 0.67 fold, *P* < 0.001) compared with vehicle (Fig. 2A). We then used Western blotting to examine nNOS protein expression in IM-FEN cells. Furthermore, when compared to vehicle or hexafluoro-honokiol, PA and LPS treatment alone increased nNOS protein expression considerably. While nNOS protein expression peaked significantly when hexafluoro-honokiol was combined with PA and LPS treatment compared to other groups (hexafluoro-honokiol, 0.880 ± 0.008 fold, *P* < 0.001; PA + LPS, 1.518 ± 0.02 fold, *P* < 0.001; Hexa + PA + LPS, 3.272 ± 0.008 fold, *P* < 0.001) (Fig. 2B). These findings suggest that hexafluoro-honokiol has a positive impact on nNOS neurons and protein expression in the complicity of stress caused by PA with LPS.

### Honokiol and hexafluoro-honokiol increased SIRT3 mRNA levels and their activity

**Figure 3:**
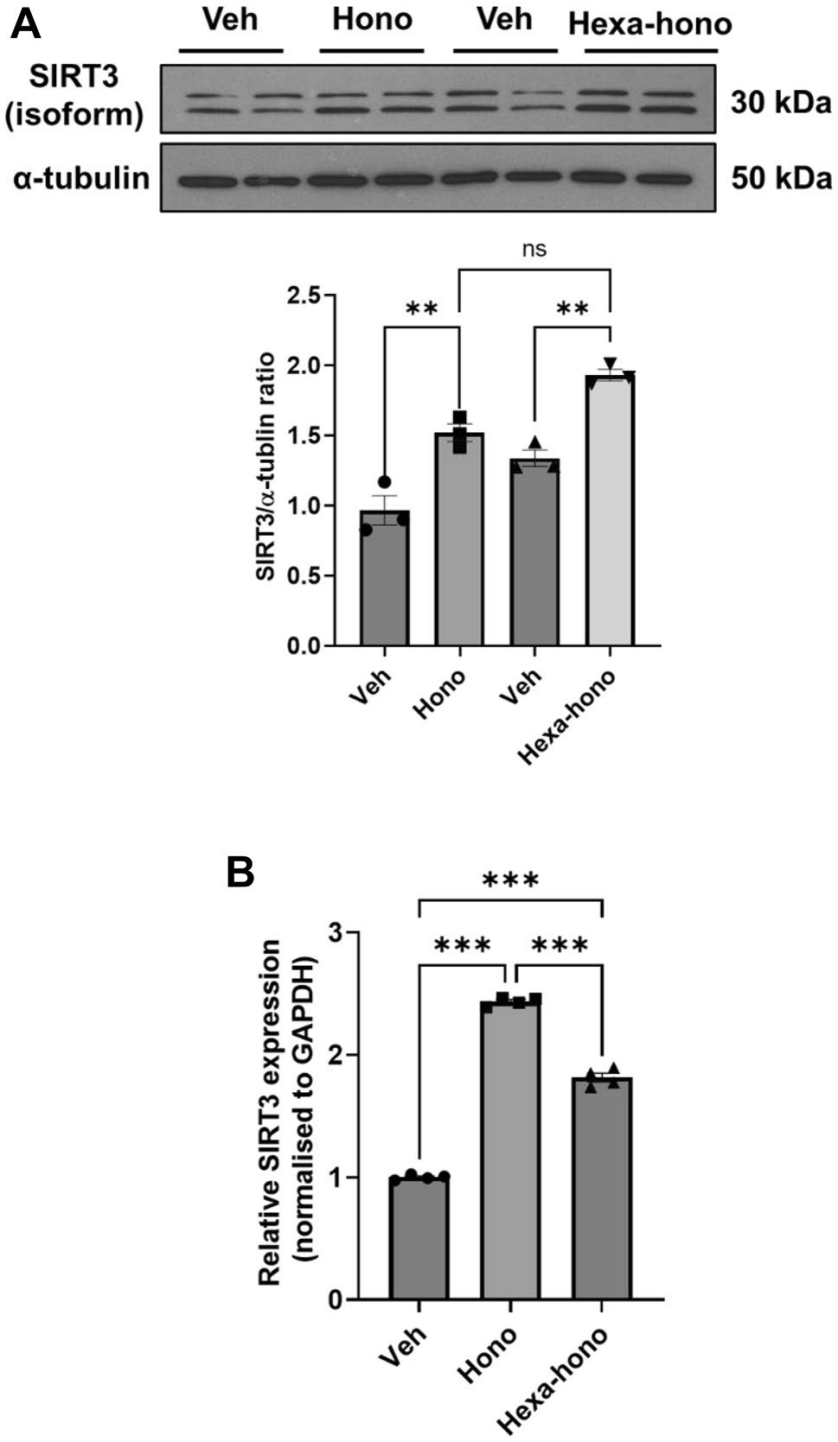
Honokiol and Hexafluoro-honokiol increase the mRNA and protein expression levels of SIRT3 in primary myenteric neurons and IM-FEN cells. (**A**) Western blot analysis was performed with protein lysates from IM-FEN cells treated with vehicle, Honokiol, or Hexafluoro, cultured for 24 h, and probed for SIRT3 protein and α-tubulin protein as a loading control. The histogram shows the SIRT3 band density normalized to α-tubulin. (**B**) Quantitative RT-PCR was done using primary myenteric neurons treated with Vehicle, Honokiol, or Hexafluoro for 24 h. The histogram represents a fold increase in SIRT3 expression in treated cells compared to vehicle treatment. The uncropped Western blot images of Fig. 3A have been provided in the supplementary file. Both protein of interest and loading control were run on a single membrane. Data are the mean ± SEM from three independent experiments. ns = non-significant; **P < 0.01; ***P < 0.001; (2-way ANOVA).

It has been previously shown, in neurodegenerative disorders, that SIRT3, a key mitochondrial deacetylase that regulates oxidative stress and is involved in mitochondrial quality control^42^, and honokiol enhance SIRT3 to promote neuroprotective activity^16,32,43^. We have examined the role of SIRT3 in the primary enteric and IM-FEN cells upon treatment with honokiol (10 μM) and hexafluoro-honokiol (10 μM) for 24 h. We found honokiol and hexafluoro-honokiol increased SIRT3 protein expression levels in IM-FEN cells (honokiol, 1.53 ± 0.05 fold, *P* < 0.01; hexafluoro-honokiol, 1.94 ± 0.12 fold, *P* < 0.001) (Fig. 3A) as well as mRNA levels in primary myenteric neurons (honokiol, 2.43 ± 0.01 fold, *P* < 0.001; hexafluoro-honokiol, 1.81 ± 0.03 fold, *P* < 0.001) relative to vehicle control (Fig. 3B). We have also found that SIRT3 significantly decreased in male and female mice fed with 12 weeks of high-fat diet (RD-M, 0.90 ± 0.02; HFD-M, 0.71 ± 0.04, *P* < 0.01) (Fig. S1A) and (RD-F, 0.99 ± 0.12; HFD-F, 0.55 ± 0.05, *P* < 0.01) (Fig. S1B) indicating the effect of high-fat diet on SIRT3 expression and a potential mechanism for HFD-induced neuronal loss that has been previously demonstrated^44^.

### SIRT3 is essential for Hexafluoro-honokiol mediated suppression of PA and LPS induced mitochondrial oxidative stress in enteric neurons

**Figure 4:**
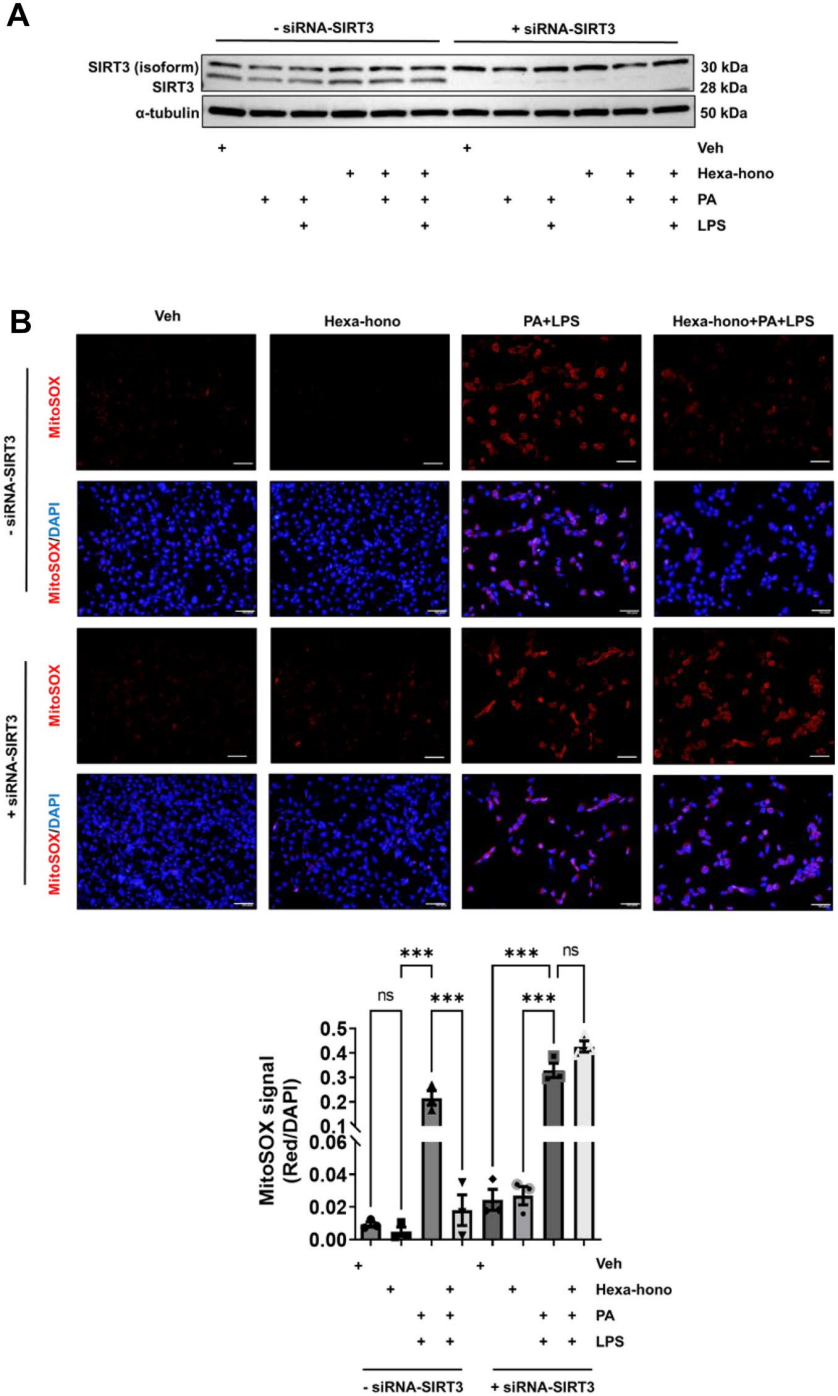
SIRT3 is required for Hexafluoro-honokiol mediated mitochondrial stress reduction in enteric neuronal cells. (**A**) Western blot analysis was performed with protein lysates from control and SIRT3 siRNA transfected IM-FEN cells that were treated with vehicle, PA, PA with LPS, Hexafluoro-honokiol, Hexafluoro-honokiol with PA, Hexafluoro-honokiol with PA and LPS, cultured for 24 h, and probed for SIRT3 protein and α-tubulin as a loading control. (**B**) Determination of mitochondrial superoxide using MitoSOX assay in IM-FEN cells treated with vehicle, Hexa, PA with LPS, hexafluoro with PA and LPS, in IM-FEN cells transfected with control or SIRT3-siRNA. The histogram represents MitoSOX positive cells/DAPI stained cells. The uncropped Western blot images of Fig. 4A have been provided in the supplementary file. Both protein of interest and loading control were run on a single membrane. Data are the mean ± SEM from three independent experiments. ns = non-significant; ***P < 0.001; (2-way ANOVA).

As seen in Fig. 3A and B, honokiol and hexafluoro-honokiol increased SIRT3 expression levels. The effect of a SIRT3 knockdown on honokiol-induced enteric neuronal differentiation was then investigated. IM-FEN cells transfected with vehicle siRNA and SIRT3 siRNA were exposed to PA or PA with LPS and treated with hexafluoro-honokiol or vehicle for 24 h. SIRT3 protein expression was reduced in cells transfected with SIRT3 siRNA (Fig. 4A). We used the MitoSOX assay to determine mitochondrial reactive oxygen species^45^ release indicated by MitoSOX signals in IM-FEN cells treated with PA with LPS, hexafluoro-honokiol in the presence of control or SIRT3 siRNA, In the vehicle siRNA transfected group, hexafluoro-honokiol played a neuroprotective role by suppressing the superoxide release induced by the treatment of PA with LPS (hexafluoro-honokiol, 0.005 ± 0.003-fold, ns; PA + LPS, 0.21 ± 0.03 fold, *P* < 0.001; Hexa + PA + LPS, 0.018 ± 0.01 fold, ns) compared to vehicle whereas, in SIRT3 siRNA transfection, hexafluoro-honokiol did not play any role in reducing the superoxide release (siRNA-SIRT3-hexafluoro-honokiol, 0.02 ± 0.005-fold, ns; siRNA-SIRT3-PA + LPS, 0.33 ± 0.03 fold, *P* < 0.001; siRNA-SIRT3-Hexa + PA + LPS, 0.42 ± 0.04 fold, *P* < 0.001) (Fig. 4B) indicating the primary importance of SIRT3 in the regulation of mitochondrial health in enteric neurons.

### Hexafluoro-honokiol prevents cell pyroptosis from palmitate and LPS-induced stress

**Figure 5:**
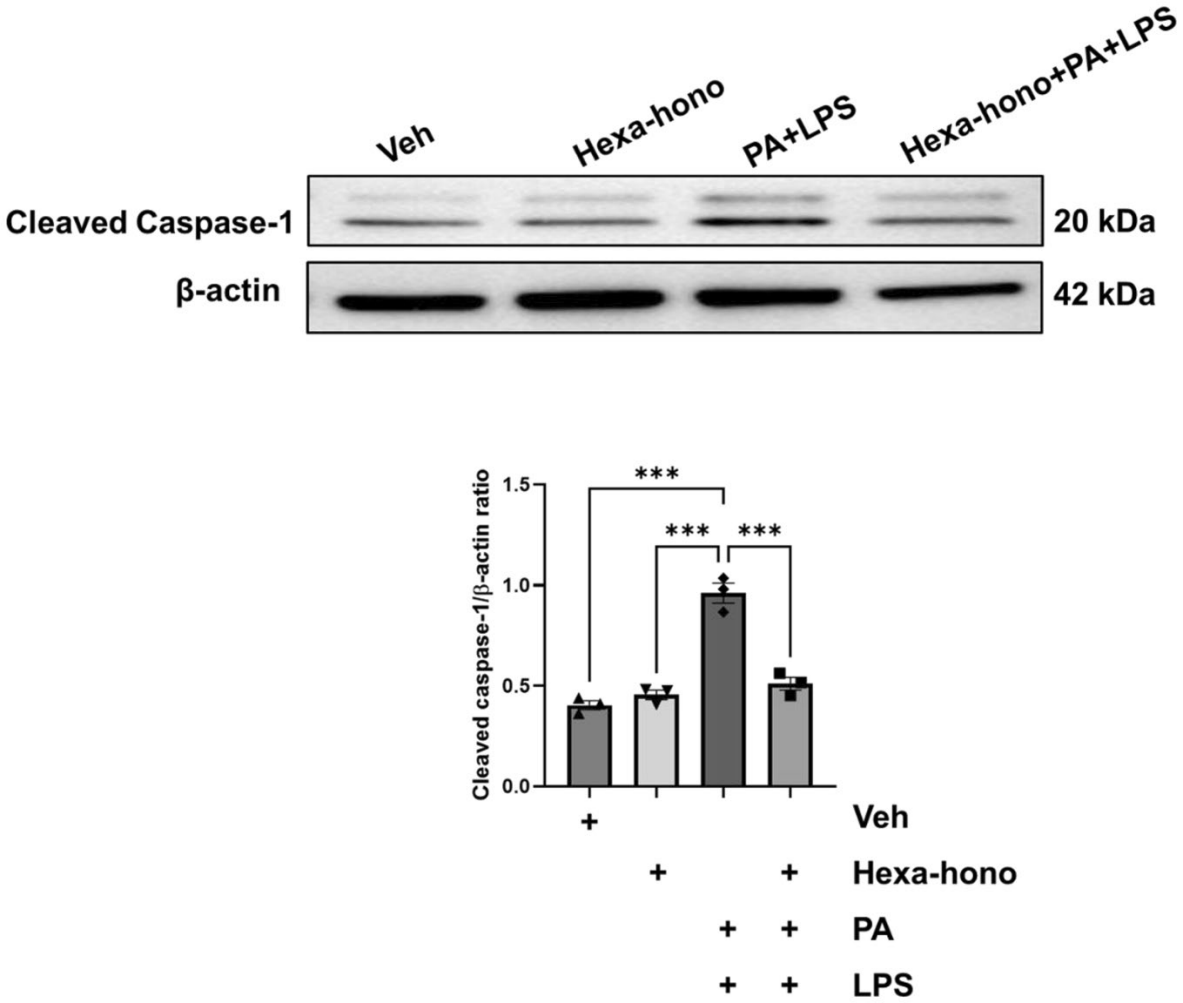
Hexafluoro-honokiol suppresses palmitate and palmitate plus LPS-induced enteric neuronal cell pyroptosis. Western blot was run with protein lysates from IM-FEN cells treated with vehicle, Hexafluoro-honokiol, PA with LPS, Hexafluoro with PA and LPS, cultured for 24 h, and probed for cleaved caspase-1 and β-actin as a loading control and mouse brain lysate was used as a positive control. The histogram shows Cleaved caspase-1 band density normalized to β-actin. The uncropped Western blot images of Fig. 5 have been provided in the supplementary file. Both protein of interest and loading control were run on a single membrane. Data are the mean ± SEM from three independent experiments. ***P < 0.001; (2-way ANOVA).

To determine if hexafluoro-honokiol mediated reduction in mitochondrial stress leads to suppression of enteric neuronal cell death we assessed neuronal pyroptosis. Pyroptosis is defined by the caspase 1-dependent generation of plasma-membrane pores, which results in pathogenic ion fluxes, cellular lysis, and the release of proinflammatory intracellular substances^46^. We have previously demonstrated PA and LPS induced neuronal pyroptosis^47^. To assess the role of hexafluoro-honokiol in the prevention of cell pyroptosis, we measured the levels of cleaved caspase-1 in IM-FEN cells that are exposed to PA with LPS and treated with hexafluoro-honokiol or vehicle for 24 h. Hexafluoro-honokiol decreased the expression of cleaved caspase-1 significantly and suppressed pyroptosis in palmitate plus LPS induced cell stress (hexafluoro-honokiol, 0.46 ± 0.03-fold, ns; PA + LPS, 0.97 ± 0.04 fold, *P* < 0.001; Hexa + PA + LPS, 0.51 ± 0.09 fold, *P* < 0.001) (Fig. 5) representing a protective role of hexafluoro-honokiol in enteric neuronal cells.

## Discussion

In this study, we have shown for the first time that honokiol and its analog, hexafluoro-honokiol, exert beneficial effects on the ENS. Previous studies have reported that honokiol protected against oxidative stress by restoring mitochondrial functions and Na^+^, K^+^-ATPase levels^48^, Honokiol also suppressed inflammation in the brains of mice with cerebral ischemia and post injury^20,49^ and it activated SIRT3 in mice with cardiac hypertrophy^16,32^. These reports demonstrate that honokiol activates SIRT3 to deacetylate downstream mitochondrial targets and preserve mitochondrial health. Reduced levels of SIRT3 have been associated with aging, disease, or poor lifestyle habits^23^. The knockdown of SIRT3 in experimental models of various diseases^23,24^ increased oxidative damage, and caused severe alterations in the acetylation status of mitochondrial proteins, further emphasizing the role of SIRT3 in maintaining mitochondrial homeostasis under stress.

**Figure 6:**
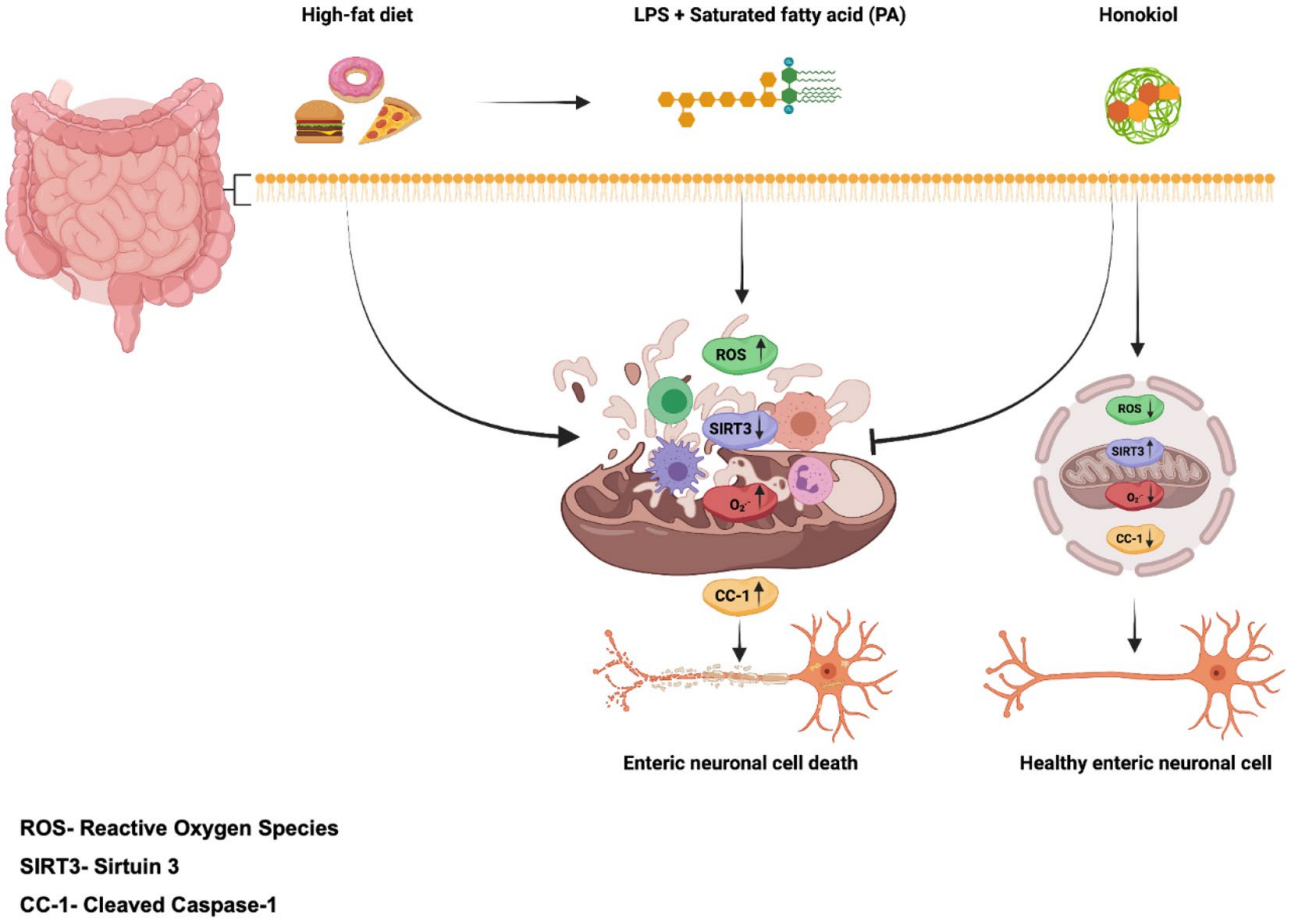
Model illustrating a potential mechanism of Honokiol/Hexafluoro mediated activation of SIRT3 and preventing neurodegeneration in enteric neurons. High-fat diets rich in saturated fatty acids like palmitic acid (PA) and endotoxins like lipopolysaccharide (LPS) can cause metabolic stress in the enteric nervous system via induced ROS, which can lead to SIRT3 depletion mitochondrial sirtuin 3 (SIRT3). SIRT3 depletion causes the generation of pyroptotic markers like cleaved caspase-1. Honokiol and hexafluoro treatment may be increasing SIRT3 expression to reduced mitochondrial ROS and suppress pyroptosis.

In our study, honokiol increased the survival and differentiation of primary myenteric neuronal cells isolated from mice. Also, honokiol notably enhanced the expression of the genes that code for the nNOS (inhibitory neurotransmitter^50^) and ChAT (excitatory neurotransmitter^50^). The ENS can be damaged by high-fat diets rich in saturated fatty acids such as PA and LPS endotoxins which are an integral part of gram-negative bacteria and produce pro-inflammatory cytokines^41^. We have previously shown that PA and LPS induce the loss of nitrergic neurons in vitro to alter the gut microbiome and affect motility in vivo^41^. Nitrergic neurons produce nitric oxide (NO), an inhibitory neurotransmitter that relaxes gastrointestinal smooth muscle^40^. PA and LPS induced oxidative stress can cause a modest upregulation of nNOS expression. Hexafluoro-honokiol significantly enhances the expression of nNOS neurons and proteins**.** Our data indicate that treatment with honokiol and hexafluoro-honokiol increases SIRT3 in mRNA and protein levels in neuronal cells (IM-FEN). Furthermore, we have also shown that honokiol and hexafluoro-honokiol are dependent on SIRT3 to exert an anti-pyroptotic effect on enteric neurons in the presence of PA and LPS. Further, blocking SIRT3 prevented hexafluoro–honokiol mediated amelioration of mitochondrial superoxide release (Fig. 6). This agrees with previous findings that showed that honokiol activates SIRT3 and suppressed ROS production reducing cellular stress^16^. Thus, SIRT3 is essential for honokiol’s activity in the ENS and could be a potential therapeutic target for neuropathies in the ENS. We have previously demonstrated that a high-fat diet causes the loss of nitrergic neurons in the colon and ileum by activating apoptosis via the caspase-3 signaling pathway, leading to ENS neurodegeneration^41^. In this study, we assessed if enteric neurodegeneration caused by PA and LPS could be suppressed by honokiol or hexafluoro-honokiol treatment. We observe that honokiol and its analog hexafluoro-honokiol suppressed pyroptosis caused by the neurotoxic insults of PA and LPS and reduced the expression of the pyroptosis marker-cleaved caspase-1 protein. Honokiol and hexafluoro-honokiol increased the expression of SIRT3 and could potentially reduce mitochondrial oxidative stress caused by PA and LPS thereby preventing pyroptosis in neurons.

SIRT3 modulates genotoxic, metabolic, and oxidative stress using different and potentially exclusive molecular mechanisms^51^. Contrary to our data, a study showed that a mice model of inflammation (DNBS-colitis) with SIRT3 deletion as well as SIRT3 inhibition did not increase oxidative damage in their myenteric neurons when compared to the wildtype mice^52^. The authors postulate that damage control in enteric neurons caused by ROS might utilize mechanisms that did not need SIRT3 as opposed to the necessity of SIRT3 in the CNS and other organs in the body to counteract ROS. In this mouse model of inflammation, the authors examined the role of SIRT3 in preventing inflammation-induced oxidative stress. The authors did not find any role for SIRT3 in preventing oxidative stress under these conditions. Our model is based on saturated fatty acids induced neuronal injury and the differences we observe are potentially related to the different stress inducers and the subsequent signaling pathways that are activated including the increase in mitochondrial reactive oxygen species. In our study, we found that SIRT3 expression was critical to protect against PA and LPS-induced injury of enteric neurons. Our results may differ from the previous study due to differences in the models and inducers of inflammation.

Potentially, a lack of SIRT3 is compensated by other sirtuins with similar transcriptional activators, substrates, and mechanistic functions such as SIRT1^53^. Like SIRT3, SIRT1 is an NAD^+^ dependent deacetylase that regulates metabolic processes by acting as an energy sensor, and is widely expressed in the nucleus of enteric neuronal cells^54^, and has shown to prevent intestinal inflammation caused by acute colitis^55,56^. Further studies are needed to examine the role of other sirtuins in the ENS.

We propose that honokiol and hexafluoro-honokiol can increase the antioxidant response of SIRT3 in the ENS during metabolic stress by promoting the transcription of SIRT3 by binding to the antioxidant response element in SIRT3 which releases antioxidants and other neuroprotective chemicals and thus counteracts oxidative damage^57^. Alternatively, honokiol may increase SIRT3 transcription directly via PGC-1α (Peroxisome proliferator-activated receptor gamma coactivator 1-alpha), a master regulator of mitochondrial energy metabolism^32^. It has been shown that PGC-1α works with Nrf2 as a transcriptional coactivator to regulate SIRT3 expression^58^. More studies on how honokiol mediates neuroprotection via Keap1/Nrf2/SIRT3-ARE signaling cascade will be useful to position it as a pharmacological intervention to treat enteroneuropathies.

In summary, our findings demonstrate an important role for honokiol and hexafluoro-honokiol in promoting enteric neuronal survival and differentiation through increased SIRT3 expression. These studies show promising effects of using honokiol or its derivative as a potential therapeutic strategy in treating ENS-related neurodegenerative disorders.

## Supplementary Information

Supplementary Information.

## Data Availability

All data obtained or analyzed throughout this study are included in the manuscript and supplementary files.
