# Supplementary material for: SIRT3 activation promotes enteric neurons survival and differentiation
Source: Sci Rep. 2022 Dec 21;12:22076. doi: 10.1038/s41598-022-26634-9 (PMC9772335; doi:10.1038/s41598-022-26634-9)
Supplement: Supplementary file 1 — Supplementary Information. [file 41598_2022_26634_MOESM1_ESM.docx]

**Supplemental Information**

**SIRT3 activation promotes enteric neurons survival and differentiation**

Arun Balasubramaniam^1,4^, Ge Li^1,4^, Anita Ramanathan^1,4^, Simon Musyoka Mwangi^1,4^, Michael C. Hart ^3,4^, Jack L. Arbiser^2,4^, Shanthi Srinivasan^1,4^

^1^Division of Digestive Diseases, Emory University School of Medicine, Atlanta, GA, United States.

^2^Department of Dermatology, Emory University School of Medicine, Atlanta, GA, United States.

^3^Division of Pulmonary Medicine, Emory University School of Medicine, Atlanta, GA, United States.

^4^Atlanta VA Medical Center, Atlanta, GA, United States.

**Supplementary materials and methods**

**Western Blotting**

C57BL/6 (8-12 weeks old, male, n=3/group and female, n=3/group) fed with RD and HFD for 12 weeks. At the end of the experiment, all mice were sacrificed, and muscle strips peeled from mouse colon were lysed in 1x Laemmli samples loading buffer (Bio-Rad, Hercules, CA) supplemented with a complete protease inhibitor cocktail (Roche Diagnostics, Mannheim, Germany) and proteins separated on Criterion TGX 4-20% gels (Bio-Rad) according to recommended procedure. Separated proteins were transferred onto Immune-Blot polyvinylidene difluoride (PVDF) membranes (Bio-Rad) according to recommended procedure. anti-SIRT3 (rabbit, #5490, 1:1000, Cell Signaling Technology, Boston, MA, USA), anti-α-tubulin (mouse, #3873S, 1:1000, Cell Signaling Technology, Boston, MA, USA). Horseradish peroxidase-conjugated anti-mouse and anti-rabbit IgG (Cell Signaling Technology) secondary antibodies were used at a 1:2000 dilution. All semi-quantitative measurement of band intensity was performed using the ImageJ analysis software (US National Institutes of Health, Bethesda, Maryland, USA).

**
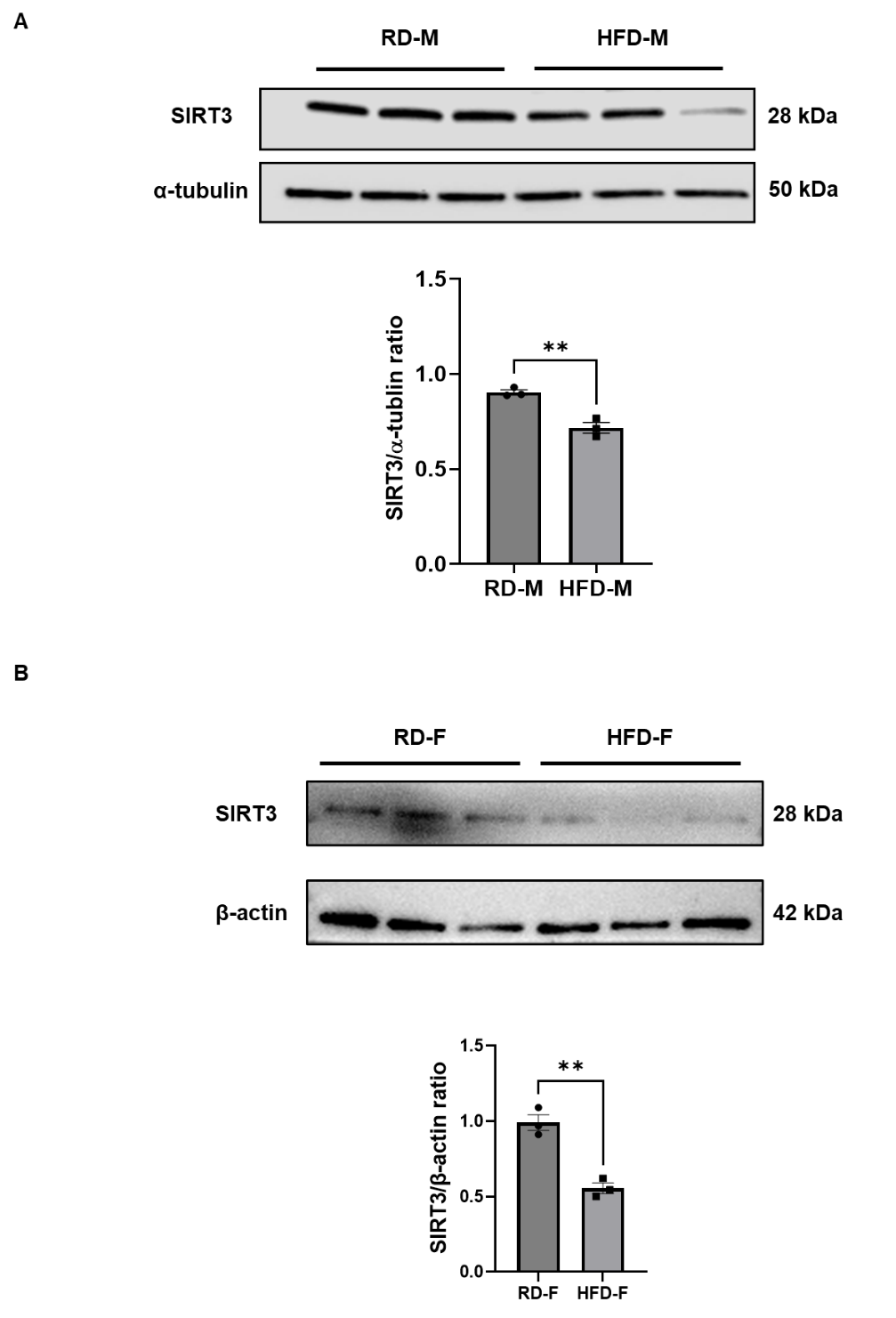
**

**Figure S1.** A) The protein lysate from the male mice group fed with 12 weeks of regular and high-fat diet was probed with SIRT3 and α-tubulin. The histogram shows the SIRT3 band density normalized to α-tubulin. B) The female mice group fed with 12 weeks of regular and high-fat diet was probed with SIRT3 and β-actin. Data are the mean ± SEM from three separate experiments. *P < 0.05; **P<0.01; (Unpaired t-test). Both protein of interest and loading control were run on a single membrane.

**Figure S2.**  A) The uncropped Western blot images of Figure S1 A. The lanes from top to bottom of each blot represent the size of the protein ladder (kDa); The protein band levels of SIRT3 & α-tubulin are demonstrated. B) The uncropped Western blot images of Figure S1 B. The protein band levels of SIRT3 & β-actin are demonstrated. Both protein of interest and loading control were run on a single membrane.

**Figure S3.** The uncropped Western blot images of Figure 2B. The lanes from top to bottom of each blot represent the size of the protein ladder (kDa); The protein band levels of nNOS & β-actin are demonstrated. Both protein of interest and loading control were run on a single membrane.

**Figure S4.** The uncropped Western blot images of Figure 3A. The lanes from top to bottom of each blot represent the size of the protein ladder (kDa); The protein band levels of SIRT3 & α-tubulin are demonstrated. Both protein of interest and loading control were run on a single membrane.

**Figure S5.** The uncropped western blot images of Figure 4A. The lanes from top to bottom of each blot represent the size of the protein ladder (kDa); The protein band levels of SIRT3 & α-tubulin are demonstrated. Both protein of interest and loading control were run on a single membrane.


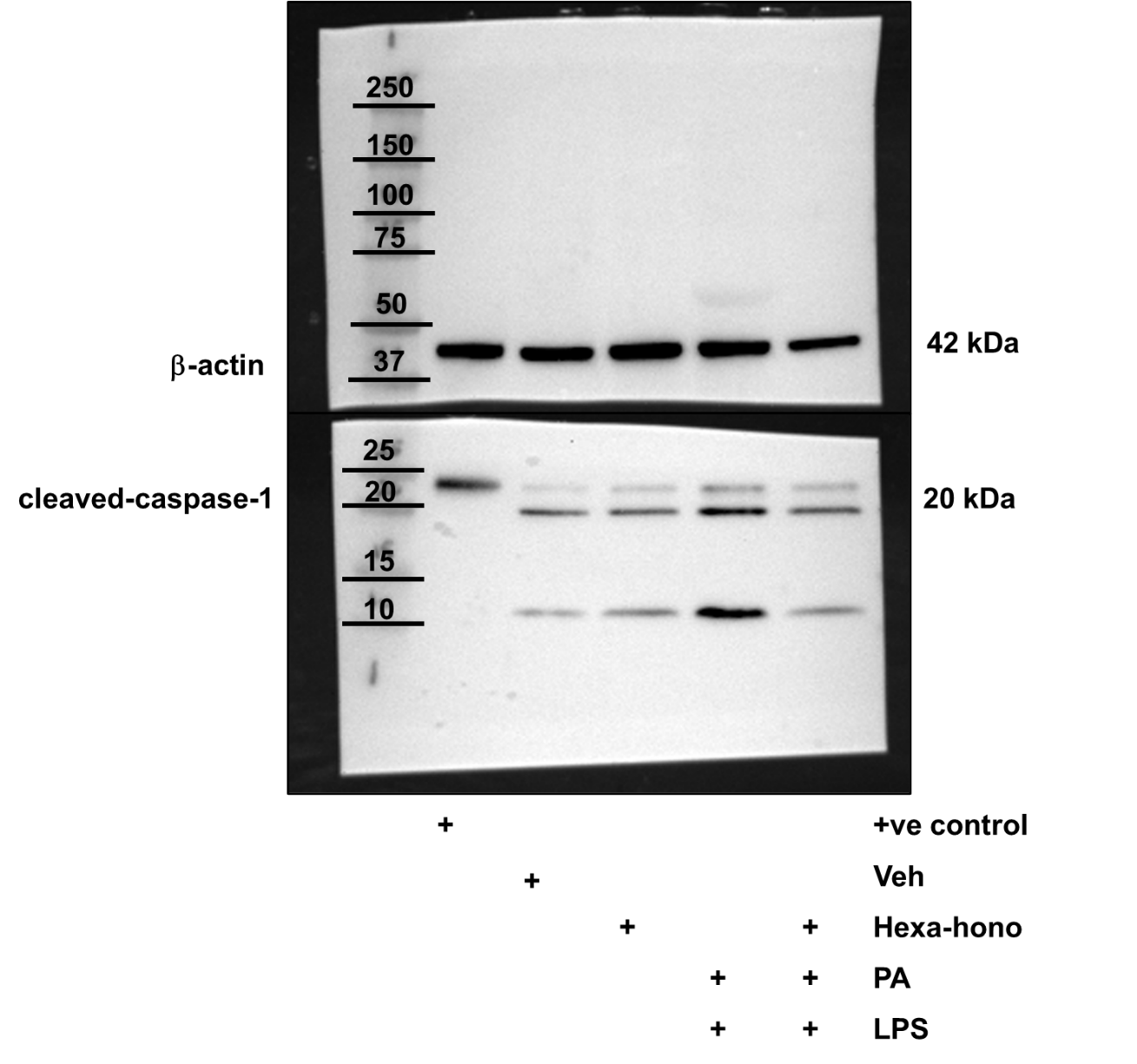


**Figure S6.** The uncropped Western blot images of Figure 5. The lanes from top to bottom of each blot represent the size of the protein ladder (kDa); The protein band levels of cleaved-caspase-1 & β-actin are demonstrated. Both protein of interest and loading control were run on a single membrane.
